# Ca^2+^/Sr^2+^ Selectivity in Calcium-Sensing Receptor (CaSR): Implications for Strontium’s Anti-Osteoporosis Effect

**DOI:** 10.3390/biom11111576

**Published:** 2021-10-24

**Authors:** Diana Cheshmedzhieva, Sonia Ilieva, Eugene A. Permyakov, Sergei E. Permyakov, Todor Dudev

**Affiliations:** 1 Faculty of Chemistry and Pharmacy, Sofia University “St. Kl. Ohridski”, 1164 Sofia, Bulgaria; dvalentinova@chem.uni-sofia.bg (D.C.); silieva@chem.uni-sofia.bg (S.I.); 2 Institute for Biological Instrumentation of the Russian Academy of Sciences, Federal Research Center ‘Pushchino Scientific Center for Biological Research of the Russian Academy of Sciences’, 142290 Pushchino, Russia; epermyak@yandex.ru

**Keywords:** calcium-sensing receptor (CaSR), Ca^2+^/Sr^2+^ selectivity, Mg^2+^, DFT/PCM calculations, CaSR agonists, osteoporosis

## Abstract

The extracellular calcium-sensing receptor (CaSR) controls vital bone cell functions such as cell growth, differentiation and apoptosis. The binding of the native agonist (Ca^2+^) to CaSR activates the receptor, which undergoes structural changes that trigger a cascade of events along the cellular signaling pathways. Strontium (in the form of soluble salts) has been found to also be a CaSR agonist. The activation of the receptor by Sr^2+^ is considered to be the major mechanism through which strontium exerts its anti-osteoporosis effect, mostly in postmenopausal women. Strontium-activated CaSR initiates a series of signal transduction events resulting in both osteoclast apoptosis and osteoblast differentiation, thus strengthening the bone tissue. The intimate mechanism of Sr^2+^ activation of CaSR is still enigmatic. Herewith, by employing a combination of density functional theory (DFT) calculations and polarizable continuum model (PCM) computations, we have found that the Ca^2+^ binding sites 1, 3, and 4 in the activated CaSR, although possessing a different number and type of protein ligands, overall structure and charge state, are all selective for Ca^2+^ over Sr^2+^. The three binding sites, regardless of their structural differences, exhibit almost equal metal selectivity if they are flexible and have no geometrical constraints on the incoming Sr^2+^. In contrast to Ca^2+^ and Sr^2+^, Mg^2+^ constructs, when allowed to fully relax during the optimization process, adopt their stringent six-coordinated octahedral structure at the expense of detaching a one-backbone carbonyl ligand and shifting it to the second coordination layer of the metal. The binding of Mg^2+^ and Sr^2+^ to a rigid/inflexible calcium-designed binding pocket requires an additional energy penalty for the binding ion; however, the price for doing so (to be paid by Sr^2+^) is much less than that of Mg^2+^. The results obtained delineate the key factors controlling the competition between metal cations for the receptor and shed light on some aspects of strontium’s therapeutic effects.

## 1. Introduction

The extracellular calcium-sensing receptor (CaSR), a member of the G protein-coupled receptor superfamily, plays a key role in regulating Ca^2+^ concentration in extracellular fluids, which (in resting conditions) are usually within the 1.1–1.3 mM range [1]. It is expressed in a variety of tissues, such as parathyroid glands, kidneys and bone cells, and has been assigned to perform an active role in maintaining skeletal homeostasis [2,3]. CaSR senses alterations in Ca^2+^ concentration in blood and tightly controls Ca^2+^ levels by modulating the synthesis of parathyroid hormone in the parathyroid glands and regulating the reabsorption of Ca^2+^ in the kidneys and bones [4]. In different bone cells (osteoblasts, osteoclast precursors and mature osteoclasts), CaSR governs vital cellular functions such as cell growth, differentiation and apoptosis [2]. The binding of the native agonist, Ca^2+^, to CaSR activates the receptor, which undergoes structural changes, thus, triggering a cascade of events along the phospholipase C and cAMP-dependent signal transduction pathways [1].

The CaSR is a homodimer with a molecular mass of 240-310 kDa [5]. A recent X-ray study of the extracellular domain of the human CaSR revealed four metal-binding sites in each monomer, one of which (Site 2) is loaded with Ca^2+^ in both the inactive and active states of the receptor, whereas, the other three sites (Site 1, Site 3 and Site 4) are populated by the cognate Ca^2+^ in the activated state only [6]. The presence of the metal-loaded Site 2 in both inactive and active states of the CaSR implies that it is an integral part of the receptor’s structure. The experiments have suggested that Site 2 provides a crucial framework for the recognition of the receptor co-activator, L-Trp [6]. Crystallographic data have also shown that the bound Ca^2+^ ions have different peak heights in the anomalous difference maps, which suggests different Ca^2+^-occupancies (and respectively, affinities) of the metal-binding sites. The Ca^2+^ ions at Sites 1 and 2 have strong peaks, suggesting that these are high-occupancy sites. The Ca^2+^ ions at Sites 3 and 4 have weaker anomalous peaks, which implies lower occupancy/metal affinity of these sites. The Ca^2+^ ion at Site 4 has the weakest peak compared with the other sites, which is consistent with the site being loaded only at elevated concentrations of the activator [6].

Site 1 is located in a loop region and comprises backbone carbonyl groups of Ile81, Ser84, Leu87 and Leu88 lining the binding pocket. The metal cation in Site 2 is directly coordinated by the side chain of Thr100 and indirectly (via a water molecule) by the side chain of Asn102. The calcium ion in Site 3 is bound in an outer-shell mode (via water molecules) to Ser302 and Ser303, whereas the side chain of Asp234 and backbone carbonyl groups of Glu231 and Gly557 surround the metal cation in Site 4. Note that these binding pockets are somewhat atypical (less crowded) for the calcium ion, which usually binds to constellations of 5 or 6 protein ligands [7,8]. The role of bound Ca^2+^ ions in activating CaSR has been found to be mostly structural: they stabilize the active state by enhancing homodimer interactions between membrane-proximal domains [6].

The CaSR can be activated by other divalent/trivalent metal cations and organic polycations as well, though with lesser effectiveness compared to that of the cognate activator [1]. The activation of the receptor by Sr^2+^ is of particular interest since it is considered as the major mechanism through which strontium (in the form of soluble salts) exerts its anti-osteoporosis effect, mostly in postmenopausal women [1,9,10]. Strontium-activated CaSR initiates a series of signal relay processes promoting both osteoclast apoptosis and osteoblast differentiation, thus strengthening the bone material. In support of this mechanism, it has been experimentally demonstrated that Sr^2+^, unlike other divalent metal cations, such as Mg^2+^, is a full agonist of CaSR with efficacy close to that of Ca^2+^ [1,10]. The rationale behind this lies in the fact that the properties of Ca^2+^ and Sr^2+^ ions are rather close. They are spherical, doubly charged, alkali-earth metal cations with similar physico-chemical properties and marked affinity toward “hard” (less polarizable) oxygen-containing ligands. Their ionic radii are also similar: 1.0/1.06 Å for Ca^2+^ and 1.18/1.21 Å for Sr^2+^ in hexacoordinated/heptacoordinated complexes, respectively [11]. The respective hydration-free energies are not very different either: −359.7 kcal/mol for Ca^2+^ and −329.8 kcal/mol for Sr^2+^ [12]. In the human body, the two metals behave similarly, exhibiting distinct bone-seeking properties [13].

The intimate mechanism of Sr^2+^ activation of CaSR is, however, not fully understood. Several outstanding questions remain to be answered. To what extent are Ca^2+^/Sr^2+^-selective metal binding sites of the activated CaSR? How effectively could the “alien” Sr^2+^ compete with the native Ca^2+^ for binding sites of the receptor? What are the key determinants of the metal affinity/selectivity of CaSR in the activated state? Why is Sr^2+^ a full CaSR agonist while other divalent cations, like Mg^2+^, are much less effective in activating the host protein?

Herein, we try to answer these questions by studying the Ca^2+^/Sr^2+^ competition in the metal-binding sites of the activated CaSR, employing density functional theory (DFT) calculations combined with polarizable continuum model (PCM) computations. The first/second-shell ligands and metal cations are treated explicitly using quantum chemical methods to account for electronic effects such as the polarization of the participating entities and charge transfer from the ligands to the metal cation, whereas the rest of the protein is represented by a continuum dielectric constant ranging from 4 (buried binding sites) to 29 (binding pockets with high solvent exposure). Such an approach allows treating the strong electrostatic interactions between the two competing metal ions and protein ligands in computing the Ca^2+^ → Sr^2+^ exchange free energies and assessing the metal’s affinity toward the coordinating ligands as accurately as possible. The findings of this work, which are consistent with available experimental data, help delineate the key factors making Sr^2+^ a potent agonist for CaSR. 

## 2. Materials and Methods

### 2.1. Models Used

**Figure 1:**
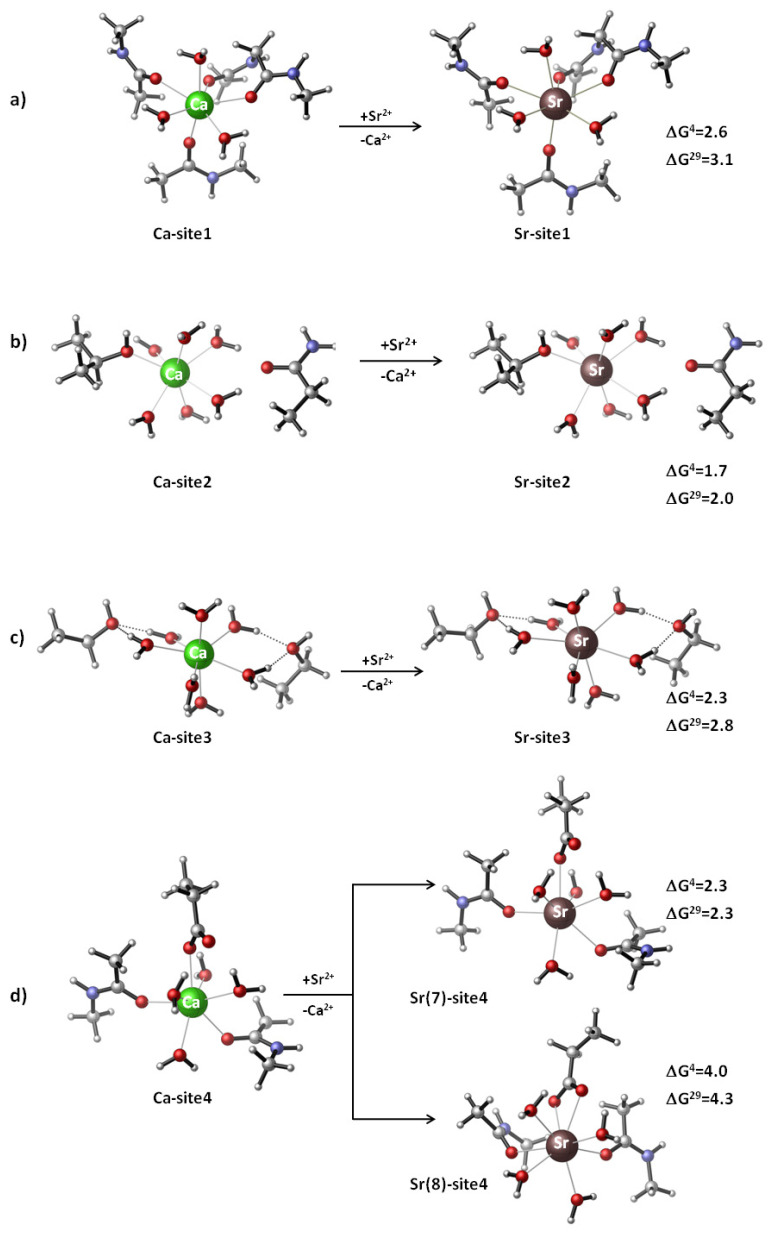
Fully optimized Ca^2+^ and Sr^2+^-loaded metal-binding sites of CaSR: (**a**) Site 1, (**b**) Site 2, (**c**) Site 3 and (**d**) Site 4 (drawn by CYLview visualization software [23]) along with the Gibbs free energies (in kcal/mol) of the Ca^2+^ → Sr^2+^ substitution.

**Figure 2:**
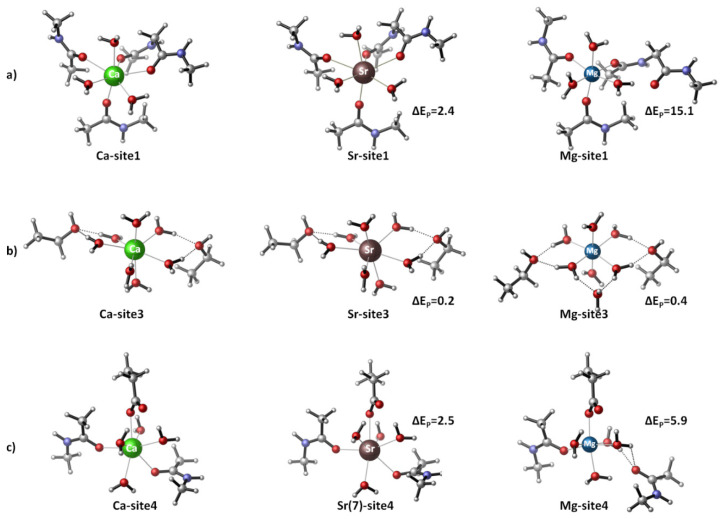
Fully optimized Ca^2+^, Mg^2+^ and Sr^2+^-loaded metal binding sites of the active-state CaSR: (**a**) Site 1, (**b**) Site 3 and (**c**) Site 4 (drawn by CYLview visualization software [23]) and energy penalties (in kcal/mol) for Sr^2+^ and Mg^2+^ structures (∆Ep = E(partial optimization) − E(full optimization); see text).

The Ca^2+^/Sr^2+^/Mg^2+^-loaded binding sites of CaSR were modeled, and their thermodynamic characteristics were evaluated. The models were built based on the X-ray structure of the Ca^2+^-bound active-state of human CaSR (PDB entry 5K5S; resolution 2.60 Å) [6]. The metal cation and its first/second shell ligands (see Introduction) were incised from the protein structure and further modified by capping the amino acid side chains at the C^α^ atom with a methyl group. Thus, the side chains of Asp^−^, Ser, Thr and Asn were represented by CH_3_CH_2_COO^−^, CH_3_CH_2_OH, CH_3_CH(OH)CH_3_ and CH_3_CH_2_CONH_2_, respectively, while the metal-coordinated backbone peptide group was modeled by N-methylacetamide (CH_3_CONHCH_3_). Since the Ca^2+^ coordination number in proteins is typically seven [14], the calcium coordination sphere in each binding site was accordingly complemented with the respective number of water molecules. The initial Ca^2+^-bound constructs created were thus subjected to geometry optimization (see below). Consequently, Ca^2+^ in the optimized structures was replaced by Sr^2+^ and Mg^2+^ and the resulting strontium/magnesium-containing constructs were fully optimized. The optimized structures of Ca^2+^, Sr^2+^ and Mg^2+^ complexes are shown in Figure 1 and Figure 2.

The competition between Sr^2+^ and Ca^2+^ can be expressed in terms of the Gibbs free energy of displacement of the native agonist, Ca^2+^, bound to CaSR, by its rival, Sr^2+^:

[Sr^2+^-aq] + [Ca^2+^-CaSR] ↔ [Sr^2+^-CaSR] + [Ca^2+^-aq] (1)

In Equation (1), [Ca^2+^/Sr^2+^-CaSR] and [Ca^2+^/Sr^2+^-aq] represent the metal cation bound to the receptor ligands inside the binding pockets and unbound outside the binding cavity (in the bulk solvent), respectively. A positive free energy value for Equation (1) implies a Ca^2+^-selective site, whereas a negative value suggests a Sr^2+^-selective one.

### 2.2. DFT/PCM Calculations

The M06-2X method [15] in conjunction with Pople’s triple zeta 6-311++G(d,p) basis set for C, H, N, O, Ca and Mg atoms, and SDD basis set/effective core potential for Sr was employed in the calculations. This combination of theoretical method/basis sets has been thoroughly calibrated and validated in our previous studies with respect to available experimental data and proven to be reliable as it properly reproduced the geometry of a series of representative metal structures [16] as well as the Gibbs free energies of metal substitution in acetate, imidazole and glycine complexes [17].

Each metal-loaded binding site was optimized in the gas phase by employing the Gaussian 09 package of programs [18]. Electronic energies, E_el_, were evaluated for each optimized complex. Subsequent frequency calculations were performed at the same M06-2X/6-311++G(d,p)//SDD level of theory to prove a local minimum on the potential energy surface—no imaginary frequency was found for any of the structures studied. The frequencies were scaled by an empirical factor of 0.983 [15] and employed to calculate the thermal energies, E_th_, including zero-point energy, and entropies, S. The differences E_el_, E_th_ and S between the products and reactants in Equation (1) were used to evaluate the metal exchange Gibbs free energy in the gas phase, ∆G^1^, at T = 298.15 K according to:

∆G^1^ = ∆E_el_^1^ + ∆E_th_^1^ − T∆S^1^ (2)

The basis set superposition error for the type of reactions modeled by Equation (1) is negligible [19] and, thus, was not considered in the present evaluations.

Metal-binding sites in metalloproteins are located in cavities/crevices of the protein structure whose dielectric properties differ from those in the bulk water [20] and behave similarly to the low-polarity solvents [21]. Thus, condensed-phase calculations were conducted in solvents mimicking the dielectric properties of buried and solvent-accessible binding sites, diethyl ether (ε = 4) and propanonitrile (ε = 29), respectively. Solvation effects were accounted for by employing the solvation model based on the density (SMD) scheme [22] as implemented in the Gaussian 09 program. In doing so, the optimized structure of each metal complex in the gas phase was subjected to single-point calculations in the respective solvent at M06-2X/6-311++G(d,p)//SDD level of theory. The differences between the gas-phase and SMD energies were used to compute the solvation free energy, ΔG_solv_^ε^, of each metal construct. The incoming Sr^2+^ and outgoing Ca^2+^ metal species were considered to be in a bulk aqueous environment (ε = 78) outside the binding pocket. Accordingly, their experimentally determined hydration free energies of −329.8 kcal/mol and −359.7 kcal/mol, respectively [12], were used in the computations. The cation exchange free energy in a protein cavity characterized by an effective dielectric constant ε was evaluated as:

∆G^ε^ = ∆G^1^ + ∆G_solv_^ε^ ([Sr^2+^-CaSR]) − ∆G_solv_^ε^ ([Ca^2+^-CaSR]) − ∆G_solv_^78^ ([Sr^2+^-aq]) + ∆G_solv_^78^ ([Ca^2+^-aq]) (3)

## 3. Results

### 3.1. Competition between Ca^2+^ and Sr^2+^ for CaSR Binding Sites

Fully optimized (without any constraints) Ca^2+^- and Sr^2+^-loaded metal-binding sites of the CaSR along with the Gibbs free energies of the Ca^2+^ → Sr^2+^ substitution are presented in Figure 1. Generally, strontium, upon metal exchange, preserves the overall shape and metal coordination number seven of the “mother” calcium site (Figure 1a–d (upper part)) but, as expected, expands the size of the metal construct by elongating the bond distances with protein ligands: the average Ca^2+^-O(ligand) bond distance is 2.358 Å, whereas the respective Sr^2+^-O(ligand) bond distance is 2.515 Å. In all cases, Sr^2+^ cannot outcompete Ca^2+^ in the binding to metal centers, and this is evidenced by positive free energies of the metal exchange ranging in the narrow limits between 1.7 and 3.1 kcal/mol (Figure 1a–d (upper part)).

The results obtained imply that, although the free energies of metal exchange are not very large, these metal-binding sites are selective for Ca^2+^ over Sr^2+^ regardless of the overall net charge of the binding pockets (0 for Sites 1, 2 and 3, and −1 for Site 4) and their solvent exposure (very similar ∆G^4^ and ∆G^29^ for buried and solventaccessible metal centers, respectively). Note that Site 2, which is occupied by Ca^2+^ in both the inactive and active states of the receptor (see Introduction), behaves very similarly to Sites 1, 3 and 4. It is not severely disrupted by the incoming Sr^2+^ (Figure 1b) and is selective for Ca^2+^/Sr^2+^ with free energies of metal exchange ranging between 1.7 (∆G^4^) and 2.0 kcal/mol (∆G^29^).

Strontium, being bulkier than calcium, may adopt greater coordination numbers in its complexes. To assess the role of the increased coordination number of Sr^2+^ on the Ca^2+^/Sr^2+^ competition, we modeled a Site 4 complex where the Asp^-^ residue changed its binding mode from monodentate (Figure 1d (upper part)) to bidentate (Figure 1d (lower part)); thus, increasing the strontium coordination number from seven to eight. The resultant free energies of metal exchange for the eight-coordinated strontium (∆G^4^/∆G^29^ = 4.0/4.5 kcal/mol; Figure 1d (lower part)) are more positive (meaning decreased strontium competitiveness) than those for the seven-coordinated strontium (∆G^4^/∆G^29^ = 2.3/2.3 kcal/mol; Figure 1d (upper part)), suggesting that a significant departing from the inherited seven-coordinated structure of the metal center is unfavorable for strontium binding to CaSR. Note that increasing the metal’s coordination number (which results in increased steric repulsion between its ligands) reflects on the metal-ligand bond distances, which become longer. Indeed, the average metal-ligand bond distance in the 7-coordinated Sr^2+^ complex in Figure 1d is 2.542 Å, whereas that of its 8-coordinated counterpart (with bidentate aspartate) is 2.579 Å. Moreover, the hydrogen bonds between the second oxygen atom of Asp^-^ and the two neighboring water molecules weaken in the 8-coordinated structure as (Asp)O is now engaged with a coordinative bond with the metal: the two hydrogen bonds (Asp)-O…H_2_O elongate from 1.756/1.737 Å in the 7-coordinated (monodentate) structure to 2.175/1.948 Å in the 8-coordinated counterpart. All these factors decrease the overall stability of the latter as compared to the former.

### 3.2. Magnesium Binding to CaSR

Magnesium, unlike the full agonist strontium, has been found to be only a partial agonist of CaSR [1,10]. Why is that? Why is strontium more effective than magnesium in activating the receptor? To shed light on these issues, we modeled and fully optimized active-state CaSR binding sites loaded with Mg^2+^. The resultant structures are shown in Figure 2, where they are compared with those optimized for the native Ca^2+^ and “alien” Sr^2+^. The inspection of the structures clearly shows that the Mg^2+^ constructs, when allowed to fully relax during the optimization process, differ quite significantly from the optimized Ca^2+^ and Sr^2+^ counterparts. Mg^2+^ adopts its stringent six-coordinated octahedral structure at the expense of detaching one backbone carbonyl ligand (Site 1 and Site 4; Figure 2a,c) or a water molecule (Site 3; Figure 2b), which have been shifted to the metal second coordination layer. At the same time, Sr^2+^, as already mentioned (see above), preserves the overall structures of the binding sites, which appear to be close to those of the “mother” Ca^2+^ constructs.

The above results were obtained by assuming flexible calcium-binding sites that allow the incoming competitor to rearrange the protein ligands lining the binding pocket in accordance with its physico-chemical preferences. In many cases, however, calcium-binding sites are known to be quite rigid, forcing the attacking metal cation to adapt to their native arrangement [7,8]. The rigidity of the binding site is often considered to be one of the key factors governing the metal selectivity in calcium-binding sites [7,8]. To study the effect of the metal-binding site rigidity on the metal affinity in CaSR, we performed partial optimizations of Sr^2+^- and Mg^2+^-loaded Sites 1, 3 and 4, where the protein ligands were frozen at their original positions in the respective Ca^2+^-coordinated structures and only the metal cations and water molecules were allowed to relax. The electronic energies evaluated (designated as energy penalty for each structure, ∆E_p_) were compared with those obtained from the respective full optimizations (summarized in Figure 1). The results demonstrate that the binding of both metals to a rigid/inflexible calcium-designed binding pocket requires an extra energy penalty for the attacker (positive numbers in Figure 2). However, the price to be paid by Sr^2+^ (a couple of kcal/mol) is much less than that of Mg^2+^ for doing so (15.1 kcal/mol for Site 1 and 5.9 kcal/mol for Site 4). Thus, it appears that Sr^2+^ has an advantage over Mg^2+^ in populating relatively inflexible CaSR binding sites.

## 4. Discussion

### 4.1. Ca^2+^/Sr^2+^ Selectivity of CaSR Binding Sites

The Ca^2+^/Sr^2+^ selectivity of the active-state CaSR binding sites was assessed by DFT calculations combined with PCM computations. The results reveal that these sites, although possessing varying metal affinities, have a different number and type of protein ligands, different overall structure and charge state, and are all selective for Ca^2+^ over Sr^2+^ (Figure 1). Thus, strontium (at therapeutic concentrations in the blood of ~0.1 mM [1]) may not be able to displace the native calcium (with plasma levels of 1.1–1.3 mM [1]) from the respective metal centers. However, the exchange free energy of ~2-3 kcal/mol between Sr^2+^ and Ca^2+^ (Figure 1) is not very high and could be surmounted (in favor of strontium) in the case of Sr^2+^ concentration increases. The literature suggests that the concentration of strontium ions present locally within the bone may significantly exceed the levels present in the blood [1,10]. Furthermore, cooperativity has been found between the CaSR agonists (Sr^2+^ and Ca^2+^) in the activation of the receptor that allows the secondary activator (Sr^2+^) to activate the receptor at relatively low concentrations of the primary agonist (Ca^2+^) [9,10]. Strontium, being a calcium mimetic species, can populate (though with lower efficiency than calcium) empty metal-binding sites in inactive CaSRs and trigger the respective cellular response.

The three binding sites of the activated receptor, regardless of their structural differences, exhibit almost equal metal selectivity if they are flexible and do not impose any geometrical constraints on the incoming Sr^2+^ (very similar ∆G^4/29^ in Figure 1a,c,d (upper part)). Rigidifying the binding pockets, however, would decrease Sr^2+^ competitiveness of Site 1 and Site 4 (making them less attractive for Sr^2+^) to a greater extent than that of Site 3, as the energy penalty to be paid by the attacking Sr^2+^ for binding to the former (2.4/2.5 kcal/mol) would be higher relative to that of the latter (0.2 kcal/mol; Figure 2).

Data analysis suggests that factors, such as the number and type of protein ligands, charge state of the binding pocket and its solvent exposure do not seem to play a significant role in governing the competition between Ca^2+^ and Sr^2+^ in CaSR. Rather, these are the intrinsic physico-chemical properties of the two competing metal species (Ca^2+^ is a better Lewis acid than Sr^2+^) and the rigidity of binding sites that orchestrates the process. Similar conclusions have been drawn on the competition between Ca^2+^ and Sr^2+^ in a representative of the EF-hand family calcium protein, parvalbumin [24].

### 4.2. Sr^2+^ as a CaSR Agonist

Strontium has been found to be a *full* CaSR agonist, though with slightly lower efficacy than the native Ca^2+^ [1,10]. Indeed, Sr^2+^, as a Ca^2+^-mimetic species, reproduces very closely the structure and overall shape of the calcium-binding sites of CaSR, which results in strontium-loaded constructs that energetically are only a few kcal/mol away from their calcium-loaded counterparts. Note that any deviation from the native calcium structure, for example, increasing the Sr^2+^ coordination number from seven to eight (Figure 1d (lower part)) or the significant rearrangement of the binding site buildup, as in the case of Mg^2+^ (Figure 2), is severely penalized. For Mg^2+^, this compromises its ability to act as an effective CaSR activator, which, as some experiments show, renders magnesium as a *partial* agonist of the receptor [1,10]. Thus, we may hypothesize that Sr^2+^ is superior to Mg^2+^ in binding/activating CaSR as it (i) better mimics the calcium-binding sites in the structure, shape and, to some extent, energetics and (ii) incurs a lower energy penalty upon fitting into calcium-designed binding pockets (see above).

### 4.3. Sr^2+^ Mechanism of Therapeutic Action as Compared to Other Metallodrugs

Other abiogenic metal species (in cationic forms), such as Li^+^ and Ga^3+^, are used in medicinal practice to fight various medical conditions such as psychiatric disorders [25], neoplastic formations and bacterial infections [26,27,28]. The competition between the non-native Li^+^ and cognate Na^+^/Mg^2+^/Ca^2+^, as well as in the case of abiogenic Ga^3+^ and native Fe^3+^, in the key biological systems involved in the respective pathogeneses, have been found to constitute the core of these metals’ therapeutic actions [29,30,31]. In both cases, the “alien” cations, Li^+^ and Ga^3+^, substitute for the native metal species (Na^+^/Mg^2+^/Ca^2+^ and Fe^3+^, respectively) in the respective metalloenzymes/receptors and, as the resulting metal constructs are either structurally altered (lithium’s effect) or redox silent (gallium therapy), they suppress the activity of overexpressed disease-related proteins. The mechanism of the Sr^2+^ anti-osteoporosis action appears to be different from those mentioned above: it does not fully substitute for the native Ca^2+^ in CaSR but rather binds and activates the receptor as it closely mimics the basic physico-chemical and structural features of the native agonist. Strontium activation of CaSR is aided by a synergistic calcium binding to the receptor [9,10] and increased Sr^2+^ concentration achieved locally within the bone microenvironment [1,10].

## Data Availability

Optimized structures of the metal complexes can be obtained from DC or TD upon request.
